# Probing the energy conversion process in piezoelectric-driven electrochemical self-charging supercapacitor power cell using piezoelectrochemical spectroscopy

**DOI:** 10.1038/s41467-020-15808-6

**Published:** 2020-05-11

**Authors:** Karthikeyan Krishnamoorthy, Parthiban Pazhamalai, Vimal Kumar Mariappan, Swapnil Shital Nardekar, Surjit Sahoo, Sang-Jae Kim

**Affiliations:** 1 Nanomaterials and System Laboratory, Major of Mechatronics Engineering, Faculty of Applied Energy System, Jeju National University, Jeju, 63243 South Korea; 2 Department of Advanced Convergence Science & Technology, Jeju National University, Jeju, 63243 South Korea

**Keywords:** Energy science and technology, Materials science, Nanoscience and technology

## Abstract

The design and development of self-charging supercapacitor power cells are rapidly gaining interest due to their ability to convert and store energy in an integrated device. Here, we have demonstrated the fabrication of a self-charging supercapacitor using siloxene sheets as electrodes and siloxene-based polymeric piezofiber separator immobilized with an ionogel electrolyte. The self-charging properties of the fabricated device subjected to various levels of compressive forces showed their ability to self-charge up to a maximum of 207 mV. The mechanism of self-charging process in the fabricated device is discussed via “piezoelectrochemical effect” with the aid of piezoelectrochemical spectroscopy measurements. These studies revealed the direct evidence of the piezoelectrochemical phenomenon involved in the energy conversion and storage process in the fabricated device. This study can provide insight towards understanding the energy conversion process in self-charging supercapacitors, which is of significance considering the state of the art of piezoelectric driven self-charging supercapacitors.

## Abstract (web summary)

Devices that are capable of energy harvesting and storage are attractive for meeting daily energy demands, however they are limited by efficiency. Here the authors fabricate a siloxene-based self-charging supercapacitor power cell and probe the piezoelectrochemical effect involved in the charging process.

## Introduction

The increasing energy requirements in day-to-day life and the diminishing fossil fuel resources worldwide result in a high demand for the development of advanced energy harvesting, conversion, and storage devices^1^. These energy devices work via an independent mechanism for harvesting (nanogenerators, solar cells), conversion (photovoltaic, optoelectronic, electrochemical conversion), and storage (batteries and supercapacitors) applications^2–4^. In view of solving the energy crisis, researchers have developed new strategies to integrate two devices (energy harvester and storage) via extrinsic or intrinsic modes to achieve a self-powered system that will be meritorious for various applications from microscale (wearable/portable electronics) to macroscale (electric transport vehicles and health monitoring systems)^5–7^. A combination of a solar cell, nanogenerator, and fuel cell with either supercapacitor or batteries via extrinsic and/or intrinsic integrated systems has been studied recently by various research groups^8–10^. Herein, intrinsic integration possesses an advantage over extrinsic integration since the latter needs a complex power management system that requires additional manufacturing cost^11^. Hitherto, intrinsically integrated energy devices that are capable of harvesting, converting, and storing electrical energy in a single device is of great interest in basic science research and product development owing to their multifunctional device design and concepts^12^.

Among the various types of intrinsically integrated energy devices reported so far, a self-charging power cell (SCPC) using the piezoelectric concept with a battery developed by Prof. Z. L. Wang and co-workers^12,13^ is of prime interest. This is due to the capability of this SCPC device to harvest electrical energy from mechanical motion via a piezoelectric polymer (PVDF) separator and store it at the battery electrode via a novel “piezoelectrochemical conversion process”^14^. Later, several steps were developed by various researchers, such as porous PVDF polymeric separators for SCPCs with improved Li-ion transfer channels over plain PVDF films^15^. Recently, Xue et al.^16^ reported the use of a solid electrolyte-incorporated porous PVDF separator to improve the interfacial energy transfer process in SCPCs. Similarly, SCPCs fabricated using supercapacitor electrodes are designated as self-charging supercapacitor power cells (SCSPCs) or piezoelectric supercapacitors were also demonstrated during this decade^17,18^. The SCSPC might possess the advantage of fast charging over SCPC due to the higher power density of supercapacitor electrodes than the battery electrodes in SCPCs. The device design of SCSPC includes a piezo-polymer separator (porous PVDF, PVDF-TrFE, natural piezoelectric separator) coated with a gel electrolyte (aqueous or ionic liquid) sandwiched between supercapacitor electrodes^17,19^. To date, pseudocapacitive electrode (MnO_2_), electric double layer capacitive electrodes (such as functionalized carbon cloth and carbon nanotubes), and intercalative pseudocapacitance materials (MoSe_2_) have been used in reported SCSPC^17–21^. However, the self-charging efficiency or metrics of SCSPC are low, which limits their direct practical applications, whereas a direct correlation between the role of the electrodes and electrolytes on the performance of SCSPC is not completely understood. More importantly, the mechanism of energy conversion and storage in an SCSPC, which can be related to the “piezoelectrochemical effect”, is based on theoretical models and not yet been addressed experimentally^16,17^. Therefore, developing strategies to understand the “piezoelectrochemical effect” might provide new insights into the mechanical to electrical energy conversion and storage process in SCSPC. This will also provide benefits for optimizing the key parameters to improve the energy conversion efficiency of an SCSPC.

From this perspective, a new siloxene-based SCSPC is fabricated using siloxene sheets as supercapacitor electrodes and electrospun siloxene-incorporated PVDF piezofibers as separators coated with ionogels. The components of siloxene SCSPC (such as siloxene-coated carbon cloth electrodes, siloxene–PVDF piezofibers, and ionogels) are constructed in the form of symmetric supercapacitors (SSCs) with high flexibility. The mechanical energy-harvesting characteristics of the siloxene–PVDF piezofiber and electrochemical energy storage properties of the siloxene SCSPC are studied independently. Furthermore, the self-charging properties of the siloxene SCSPC via applying a compressive force are studied that shows their ability to convert and store energy. More importantly, piezoelectrochemical spectroscopy (PECS) measurements are performed in this work to directly probe the “piezoelectrochemical effect” involved in the self-charging process of a siloxene SCSPC. The PECS measurements of the siloxene SCSPC are an essential step in the ongoing research on SCSPC and provide significant findings on the fundamental understanding of the piezoelectrochemical energy conversion and storage process.

## Results

### Illustrating the fabrication process of siloxene SCSPCs

**Fig. 1:**
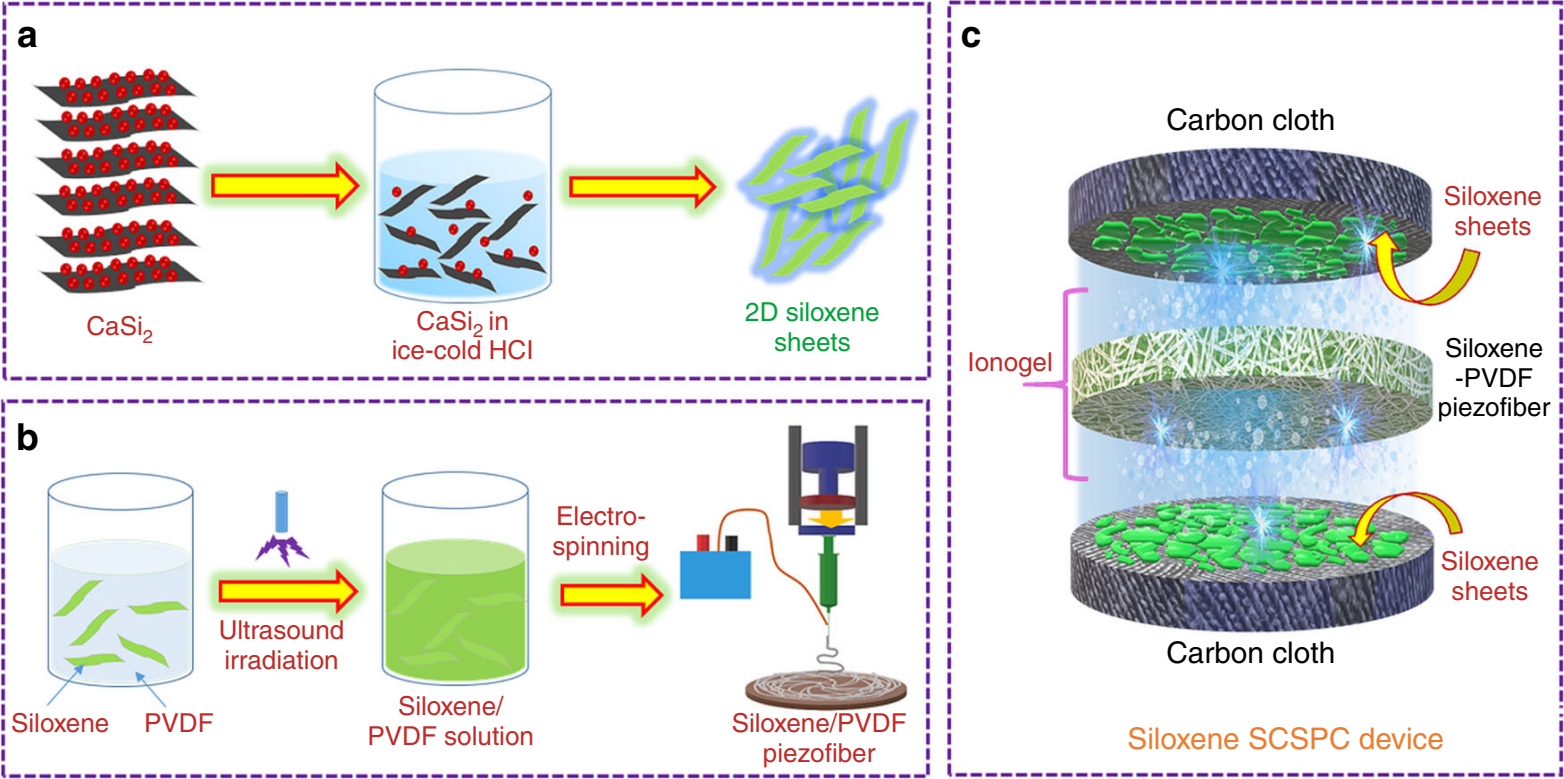
Schematic representation of steps involved in the fabrication of siloxene SCSPC. **a** represents the preparation of siloxene sheets via topochemical deintercalation of calcium from CaSi_2_ in the presence of ice-cold HCl solution, **b** represents the fabrication process involved in the electrospinning of siloxene/PVDF piezofibers, and **c** indicates the fabrication of a siloxene SCSPC device using siloxene sheets-coated carbon cloth as two symmetric electrodes and electrospun siloxene–PVDF piezofibers impregnated with ionogel electrolyte as the separator.

Figure 1 displays the graphical representation of the overall process involved in the fabrication of siloxene SCSPC. The two-dimensional (2D) siloxene sheets were synthesized through a topochemical reaction (Fig. 1a) of CaSi_2_ with ice-cold HCl for 4 days^22,23^. This process leads to the deintercalation of calcium ions with partial oxidation of Si sheets, which finally results in the formation of siloxene sheets^24^. A fine dispersion of siloxene sheets in the PVDF solution was obtained via an ultrasound irradiation process^25^, and an electrospinning process^17^ was used for the preparation of the siloxene–PVDF piezofibers, as shown in Fig. 1b. The structure of siloxene SCSPC is provided in Fig. 1c, which utilizes the siloxene-coated carbon cloth as two symmetric electrodes separated by an ionogel-coated electrospun siloxene–PVDF piezofiber separator.

### Characterization of the prepared 2D siloxene sheets

**Fig. 2:**
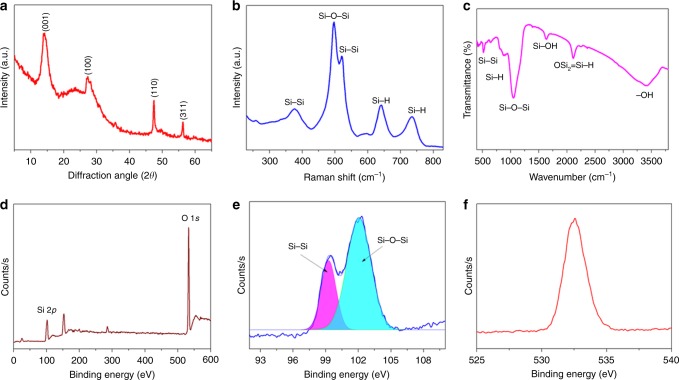
Physicochemical characterization of siloxene sheets. **a** X-ray diffraction spectrum of siloxene sheets, **b** laser Raman spectrum of siloxene sheets, **c** Fourier transformed infrared spectrum of siloxene sheets, **d** XPS survey spectrum of siloxene sheets, **e** Si 2*p*, and **f** O 1*s* core-level spectra of siloxene sheets.

Figure 2 depicts the physicochemical characterization of the prepared 2D siloxene sheets. The X-ray diffraction pattern of the 2D siloxene sheets (Fig. 2a) shows the existence of broad diffraction peaks at 13.5° and 27°, which indicate the removal of calcium from CaSi_2_ (XRD pattern of CaSi_2_ is provided in Supplementary Fig. 1) and the formation of siloxene sheets^24,26^. The laser Raman spectrum of the siloxene sheets (Fig. 2b) shows major bands at 497 and 521 cm^−1^, which corresponds to the Si–O–Si and Si–Si vibrations of the oxygen interconnected Si_6_ rings present in the hexagonal silicon frameworks of siloxene sheets^23,27^. A band located at 375 cm^−1^ arises from the vibration of Si–Si bands, whereas the other bands located at 641 and 731 cm^−1^ correspond to the Si–H vibrations in siloxene sheets^27^. The FT-IR spectrum of siloxene sheets (given in Fig. 2c) shows the existence of distinct bands at 1050 cm^−1^ (Si–O–Si networks) and 2150 cm^−1^ (O–Si_2_≡Si–H), indicating that the prepared siloxene is Kautsky-type (Si_6_ rings interconnected via Si–O–Si bridges in the Si planes as given in Supplementary Fig. 2)^23,24^. The bands centered at 525, 880, and 1632 cm^−1^ arise from the vibrations of the Si–Si, Si–H, and Si–OH groups, respectively, present in the siloxene sheets^26^. The X-ray photoelectron survey scan of the siloxene sheets (shown in Fig. 2d) highlights the presence of the Si 2*p* state (at 100 eV) and O 1*s* state (at 530 eV). The core-level spectrum of the Si 2*p* state (Fig. 2e) can be convoluted into two peaks as follows: (i) the peak at 99 eV corresponds to the Si–Si states and (ii) the peak at 102.5 eV is due to the Si–O–Si states present in siloxene sheets^26^. Figure 2f represents the O 1*s* spectrum of siloxene sheets, which originated from the oxygenated groups present in the siloxene sheets. The determined O/Si atomic ratio of the siloxene sheets from the XPS analysis is approximately 1.25; this value closely matches previous reports^23,26^.

**Fig. 3:**
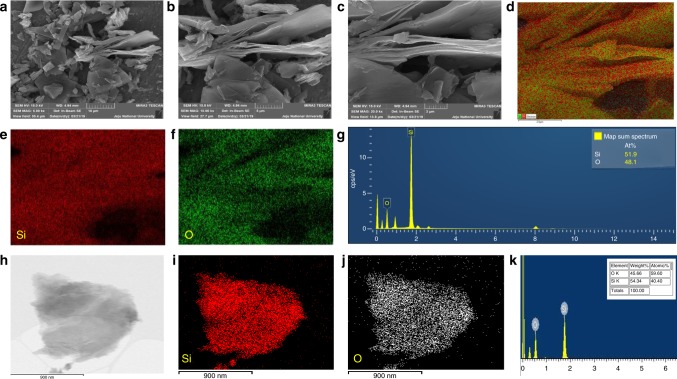
Morphological and elemental analysis of siloxene sheets. **a–c** Field emission scanning electron micrographs of the siloxene sheets. **d** Elemental mapping analysis showing the overlay map and **e–f** EDS mapping of Si and O present in the siloxene sheets; **g** EDS spectrum of Si and O present in siloxene sheets; **h** high-resolution transmission electron micrograph of individual siloxene sheets; **i**, **j** EDS mapping of Si, and O elements present in the siloxene sheets; and **k** elemental composition spectrum of Si and O present in siloxene sheets.

Figure 3 presents the morphological and elemental composition analysis of siloxene sheets using a field emission scanning electron microscope (FE-SEM) and high-resolution transmission electron microscope (HR-TEM) with elemental mapping analysis. Figure 3a–c presents the FE-SEM micrographs of the siloxene sheets (recorded under different magnifications) that clearly reveal the formation of sheet-like nanostructures of siloxene via the topochemical reaction^22,27^. The lateral dimension of the siloxene sheets is not uniform (varies from 1 to 2 μm), which might be attributed to the irregular size of the CaSi_2_ precursor used in the preparation of siloxene. Figure 3d–f represents the overlay map and Si and O elemental maps of the siloxene sheets, which shows the homogeneous distribution of Si and O atoms in the siloxene sheets. The EDS spectrum given in Fig. 3g shows that the O/Si ratio of the siloxene sheets is approximately 0.93. Figure 3h–k represents the HR-TEM micrograph of an individual siloxene sheet with a lateral size in the range of 1.2 × 1.2 μm. The elemental maps (Fig. 3i–j) indicated that the Si and O atoms are homogeneously distributed over the entire surface of the siloxene sheet, and the corresponding O/Si atomic ratio (Fig. 3k) is approximately 1.47 (analyzed using the Cliff–Lorimer ratio method)^26^. These studies confirmed the formation of 2D siloxene sheets from the topochemical reaction.

### Characterizing the electrospun siloxene–PVDF piezofiber

**Fig. 4:**
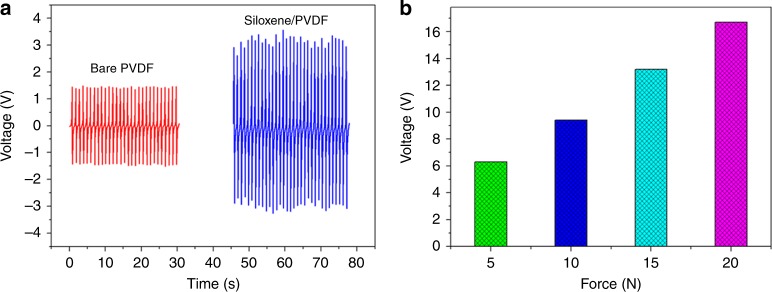
Mechanical energy-harvesting properties of siloxene–PVDF piezofibers. **a** Voltage outputs of the bare PVDF and siloxene–PVDF piezofibers subjected to an applied compressive force of 5 N. **b** Effect of applied compressive forces on the voltage output characteristics of siloxene–PVDF piezofibers subjected to various levels of applied compressive forces.

The FE-SEM micrographs of the electrospun siloxene–PVDF piezofiber obtained at low and high magnification are provided in Supplementary Fig. 3a,b. They show the presence of siloxene–PVDF fibers with a width of 50 nm with porous structures, which is an essential design consideration for ion-transportation channels in supercapacitors^28^. The elemental mapping and EDS spectrum (Supplementary Fig. 3c–h) of the siloxene–PVDF piezofibers indicate the homogeneous distribution of silicon atoms (from the siloxene sheets) in the PVDF matrix of the fibers. Figure 4a summarizes the mechanical energy-harvesting properties of the electrospun siloxene–PVDF piezofiber in comparison with the bare PVDF fiber obtained at a compressive force of 5 N. The mechanism of energy harvesting from bare PVDF fibers (with an output voltage of 3 V) is due to the piezoelectric properties of PVDF^29^. The incorporation of siloxene sheets in the PVDF matrix results in an output voltage of  ~6.5 V, which is higher than that of bare PVDF, thus indicating enhanced mechanical energy-harvesting properties. The improved energy-harvesting properties of the siloxene–PVDF piezofiber are due to the enhancement in the dipole alignments in the PVDF by the incorporation of siloxene sheets (as seen in the laser Raman spectrum given in Supplementary Fig. 4). This finding is in good agreement with earlier studies on the enhanced mechanical energy-harvesting properties of PVDF due to the incorporation of silicon and 2D materials as a filler^30,31^. The mechanical energy-harvesting properties of the siloxene–PVDF piezofiber subjected to various applied compressive forces are provided in Supplementary Fig. 5a. Figure 4b depicts the effect of various levels of compressive force on the energy-harvesting properties of the siloxene–PVDF piezofibers. The output voltage of siloxene–PVDF piezofibers has increased from ~6.5 to 16.4 V with a linear increase in the applied compressive force from 5 to 20 N, thus indicating their ideal mechanical energy-harvesting properties^32^. The ability of the siloxene–PVDF piezofibers as efficient piezoelectric nanogenerators to charge commercial capacitors is provided in Supplementary Fig. 5b. These studies validate the significance of electrospun siloxene–PVDF piezofibers for use as a piezo-polymeric separator in SCSPC.

### Energy storage performances of siloxene SCSPCs

**Fig. 5:**
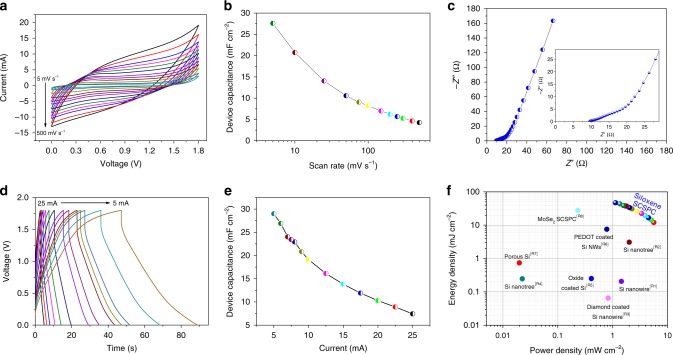
Electrochemical energy storage properties of siloxene SCSPC. **a** Cyclic voltammetric profiles of the siloxene SCSPC recorded with different applied scan rates from 5 to 500 mV s^−1^. **b** Effect of applied scan rates on the specific device capacitance of siloxene SCSPC. **c** Electrochemical impedance spectroscopic analysis of the siloxene SCSPC in the form of a Nyquist plot and the inset in **c** presents the enlarged portion of the high-frequency region. **d** Galvanostatic charge–discharge profiles of the siloxene SCSPC recorded using different applied current ranges. **e** Effect of discharge current ranges of the specific device capacitance of siloxene SCSPC, and **f** energy–power performance metrics of the siloxene SCSPC provided in the form of Ragone plot. The references given in the inset of **f** are provided in Supplementary Table 1.

The electrochemical energy storage properties of the fabricated siloxene SCSPC was evaluated using a series of experimental techniques, such as cyclic voltammetry (CV), electrochemical impedance spectroscopy (EIS), galvanostatic charge–discharge (CD) analyses, and long-term stability tests. Figure 5a shows the CV profiles of the siloxene SCSPC recorded over the operating voltage window (OVW) of 1.8 V using different applied scan rates (from 5 to 500 mV s^−1^). The presence of quasi-rectangular-shaped CV profiles indicated that the mechanism of charge storage at the siloxene electrode is due to ion-intercalation pseudocapacitance^23^. There is no observation of any redox peaks in the CV profiles, thus confirming that no Faradaic process was involved in the charge-storage nature of siloxene SCSPC. With an increase in scan rate from 5 to 500 mV s^−1^, the current range of the CV profiles is increased without any distortion in the rectangular shape, which highlights the good capacitive properties^33^. Figure 5b presents the effect of the applied scan rate on the device capacitance of the siloxene SCSPC, which possesses a high device capacitance of 27.58 mF cm^−2^ at a low scan rate of 5 mV s^−1^. Figure 5c represents the EIS data of the siloxene SCSPC device in the form of a Nyquist plot (plot of real versus imaginary component of the impedance). The Nyquist plot of the siloxene SCSPC device shows the presence of three well-defined regions, the low-, medium-, and high-frequency regions, which directly relate to the frequency-dependent synchronous-, asynchronous-, and noncharging behavior of the siloxene SCSPC^34^. From the inset of Fig. 5c, the equivalent series resistance (ESR) of the siloxene SCSPC is found to be 9.7 Ω. This value is relatively higher than that of the siloxene supercapacitor using liquid TEABF_4_ electrolyte^23^, which is due to the low ionic conductivity of the ionogel compared to that of the liquid electrolyte^35^. The observation of a quasi-semicircle such as an arc in the high-frequency region is due to the charge-transfer resistance (*R*_ct_ of 4 Ω) of the siloxene SCSPC^33^. The low-frequency region shows the evidence of a straight line (Warburg line) that runs almost parallel to the *y-*axis, which indicates the charging region of the siloxene SCSPC as a result of electrolyte ions diffusion into the electrode surface^36^. The Bode phase angle plot of siloxene SCSPC (see Supplementary Fig. 6) shows that the phase angle at the low frequency (0.01 Hz) is approximately −69°, thus revealing the pseudocapacitive behavior of the electrode^37^.

Figure 5d represents the CD profiles of the siloxene SCSPC device recorded using different applied current ranges over an OVW of 1.8 V. The presence of slopped symmetric triangular-shaped CD profiles of siloxene SCSPC revealed the pseudocapacitive (intercalation type) charge-storage nature^23^. The effect of applied currents on the device capacitance of the siloxene SCSPC is summarized in Fig. 5e. The device capacitance increased linearly with a decrease in the applied current range, and a high device capacitance of approximately 28.98 mF cm^−2^ was obtained for the siloxene SCSPC from the CD profiles recorded using a current of 5 mA. Figure 5f depicts the energy–power performance metrics of the siloxene SCSPC device in the form of a Ragone plot. It showed that the siloxene SCSPC possesses a high energy density of 46.97 mJ cm^−2^ at a corresponding power density of 1.12 mW cm^−2^ determined from the CD profile recorded using an applied current of 5 mA. With an increase in the applied current range from 5 to 25 mA, the siloxene SCSPC device retains an energy density of 12.03 mJ cm^−2^, whereas the power density increases up to 5.62 mW cm^−2^. Supplementary Table 1 shows that the performance metrics of the siloxene SCSPC are comparatively superior to many of the reported silicon-based SSCs. The mechanical flexibility of the siloxene SCSPC was examined using CV analysis recorded by subjecting the device to bending conditions, as seen in Supplementary Fig. 7a. There were no significant changes in the CV profiles of the siloxene SCSPC recorded at the bent state in comparison to those of the normal state (Supplementary Fig. 7b). The retention of device capacitance (*C*/*C*_ο_) obtained at bent state (*C*) to that of normal state (*C*_ο_) is approximately 1.07, which highlights the mechanical stability of the siloxene SCSPC. Supplementary Figure 8 presents the long-term cyclic stability of the siloxene SCSPC device over 5000 continuous cycles of CD recorded using an applied current of 10 mA. After 5000 repetitive cycles, the siloxene SCSPC device retains a device capacitance of approximately 85% of its initial capacitance, indicating its superior electrochemical stability.

### Self-charging performances of siloxene SCSPCs

**Fig. 6:**
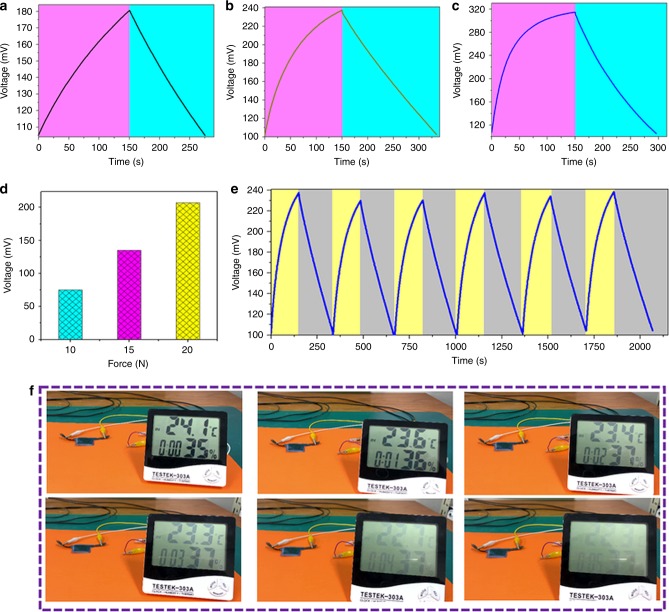
Mechanically driven self-charging properties of the siloxene SCSPC. **a–c** Self-charging properties of the siloxene SCSPC subjected to various levels of mechanical force of 10, 15, and 20 N. **d** Effect of applied compressive forces on the self-charging performance of the siloxene SCSPC. **e** Stability of the self-charging properties of the siloxene SCSPC subjected to a continuous compressive force of 15 N. **f** Practical application of the siloxene SCSPC capable of powering a multifunctional electronic display.

Figure 6 represents the self-charging behavior of the siloxene SCSPC with respect to various levels of applied compressive forces. When a compressive force of 10 N is applied on the surface of the siloxene SCSPC, the voltage of the siloxene SCSPC rises from 105 to 180 mV within 250 s, as shown in Fig. 6a, which demonstrates its self-charging properties via mechanical deformation. After this mechanically induced self-charging process, the siloxene SCSPC is allowed to discharge to its initial state within 100 s using a discharge current of 10 μA. Figure 6b, c represents the self-charging properties of the siloxene SCSPC subjected to applied compressive forces of 15 and 20 N, followed by the discharging using a constant current of 10 and 20 μA, respectively. Interestingly, the voltage of the siloxene SCSPC increases from 101 to 237 mV and 107 to 314 mV within 250 s when subjected to forces of 15 and 20 N, respectively. Figure 6d shows the self-charging performance of the siloxene SCSPC, which indicated that the device could charge up to 75, 136, and 207 mV (within 250 s) for 10, 15, and 20 N of compressive forces, respectively. The increase in the self-charging voltage of the siloxene SCSPC with an increase in the levels of applied force is due to the piezoelectric effect of the electrospun siloxene–PVDF piezofibers, which generates a greater electrical output with increasing mechanical force^16,17^. Supplementary Figure 9 presents the self-charging properties of the siloxene SCSPC fabricated using bare electrospun PVDF piezofibers as the separators. When subjected to an applied compressive force of 20 N, this device was able to self-charge up to 141 mV (from 118 to 259 mV), and this value is lower than that of siloxene SCSPC with siloxene–PVDF piezofibers as separators (Fig. 6c). This is due to the better mechanical to electrical energy conversion properties of siloxene–PVDF piezofibers than bare PVDF piezofibers, as seen in Fig. 4a. Supplementary Figure 10 shows the effect of the applied frequency of the compressive force (20 N) on the self-charging performance of the siloxene SCSPC. When the frequencies were 2, 1, and 0.5 Hz under an applied compressive force of 20 N, the self-charging performances of the siloxene SCSPC were found to be 207, 102, and 59 mV, respectively. The self-charging capacitance of the siloxene SCSPC is approximately 3.62 mF cm^−2^ (based on the calculation from discharge curve^20^) and was obtained under compressive force of 20 N. Figure 6e represents the repetitive self-charging process of the siloxene SCSPC over six consecutive cycles, which shows that outputs of the siloxene SCSPC are almost similar (130 ± 5 mV) during different cycles, thus ensuring its superior electromechanical stability. The self-charging performance metrics of the siloxene SCSPC are also compared with reported works on SCSPC so far. The performance of the siloxene SCSPC (207 mV within 250 s) is higher (based on the voltage outputs) than those of the MnO_2_ SCSPC (110 mV within 300 s)^18^, carbon cloth SCSPC (100 mV within 40 s)^20^, CNT SCSPC (70 mV within 40 s)^21^, and asymmetric supercapacitor-based SCSPC (151 mV within 80 s)^19^. The superior performance metrics might be due to the use of high-power siloxene electrodes compared to the electrodes used in the reported SCSPCs^23^. However, the time required to reach the maximum voltage (self-charging) is longer for the siloxene SCSPC than other reported ones, which might be due to the lower ionic conductivity of the ionogel (in this work) than an aqueous gel (in reported SCSPC)^38^. Considering the works undertaken on SCSPC using ionic liquid electrolytes until now, only two articles are available in the literature, which are also compared in this work. The performance of the siloxene SCSPC is lower than that of MoSe_2_ SCSPC (600 mV)^17^ and higher than that of PEDOT: PSS SCSPC (152 mV)^39^. This might be correlated with the difference in electrical conductivity and charge-storage nature of the electrode materials used in these SCSPC. This comparative analysis of siloxene SCSPC to that of reported SCSPC suggested that the electrode material, electrolyte, separator, and cell design are some of the parameters that play a key role in the self-charging metrics of an SCSPC. Figure 6f shows the practical applications of fully charged siloxene SCSPC to power up a multifunctional electronic display (MED) over 5 min. The parameters such as temperature, time, and relative humidity in the MEDs were visible over the 5 min of duration, demonstrating the practical applicability of fabricated siloxene SCSPC.

### Working mechanism of siloxene SCSPCs

**Fig. 7:**
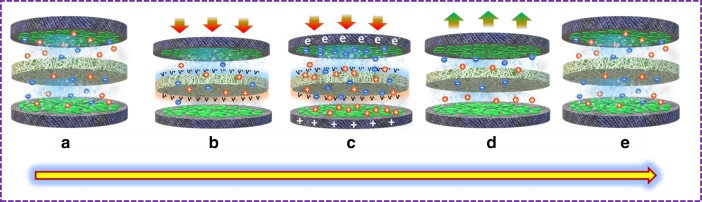
Schematic illustration of the working mechanism of the siloxene SCSPC device. **a** The initial state of the siloxene SCSPC device with no applied compressive force. **b** With an applied compressive force to the siloxene SCSPC device, the siloxene/PVDF creates a piezoelectric potential that drives the electrolyte ions towards the electrode surface. **c** An equilibrium state has been reached between the piezoelectric potential created and the electrochemical reaction of the siloxene SCSPC device. **d** State of siloxene SCSPC when the compressive force is stopped, i.e., the disappearance of piezoelectric potential and leads to attaining the equilibrium state. **e** State of siloxene SCSPC after completion of one self-charging cycle.

To date, the mechanism of the mechanically driven self-charging properties of SCSPC has been discussed based on the piezoelectrochemical process; however, the mechanism is still unclear^16,18^. The working mechanism of the siloxene SCSPC can be explained via the piezoelectrochemical process that occurred at the surface/interface between the siloxene electrode and siloxene–PVDF piezofiber, as shown in Fig. 7. In the initial state, the siloxene SCSPC is at discharged state (as shown in Fig. 7a), with two siloxene electrodes separated by the ionogelled siloxene–PVDF piezofiber in which the electrolyte ions (TEA^+^ and BF_4_^−^) are homogeneously distributed throughout the entire space inside the cell. Here, the ionogelled siloxene–PVDF piezofiber is in intimate contact with the symmetric siloxene electrodes; therefore, they can easily access the piezopotential generated from the piezofiber subjected to compressive force. When subjected to an applied compressive force, both the positive and negative piezopotential generated by the piezofibers will be accessed by the two siloxene electrodes, i.e., the positrode and negatrode, as shown in Fig. 7b. This leads to piezopotential-driven electrolyte ion migration towards the siloxene electrode (i.e., movement of TEA^+^ and BF_4_^−^ ions towards the negative and positive electrodes) via the combination of (i) physical ion-adsorption kinetics and (ii) migration of ions via ion-conducting pathways through the ionogelled siloxene–PVDF piezofiber (as shown in Fig. 7c)^40^. Notably, PVDF separators are well-known ionic conductors for TEABF_4_ ions^36^; therefore, PVDF is adequately used as a matrix/base polymer for ionogels (which is known for their mechanoionic and piezoionic properties) and binders for supercapacitor electrodes^17^. The distribution of positive and negative ions with increasing concentrations around the two siloxene electrodes results in the formation of a potential difference causing the siloxene SCSPC to self-charge (Fig. 7c). During the self-charging process of the siloxene SCSPC, the electrolyte ions will be adsorbed and/or intercalated at the siloxene electrodes, and this process will not be disturbed by the piezopotential across the siloxene–PVDF piezofiber. The potentials of the two siloxene electrodes in the SCSPC increase in opposite directions during the self-charging process (self-charging capacitance) and will reach up to a certain operating voltage until the siloxene electrode reaches a new chemical equilibrium. At this stage, the distribution of electrolyte ions equalizes the generated piezopotential, which stops the ion migration process across the interface between the electrolyte and two siloxene electrodes in the SCSPC, thus resulting in the termination of the self-charging process. Once the applied compressive force is removed (Fig. 7d), the piezoelectric properties of the electrospun siloxene–PVDF piezofiber disappear, which leads to the redistribution of the electrolyte ions to maintain the equilibrium state (Fig. 7e). When the siloxene SCSPC is again subjected to compressive force, the repetition of self-charging process is occurred. This is the overall mechanism underlying the conversion of mechanical into electrochemical energy in the siloxene SCSPC via the piezoelectrochemical phenomenon. Overall, the piezoelectric effect caused by the siloxene–PVDF piezofiber causes the positive and negative ions to drift towards the negatrode and positrode in the siloxene SCSPC, which results in the self-charging process^17^. The proposed self-charging mechanism of the siloxene SCSPC can be explained using Nernst’s theory^41,42^, which relates the electrode potential of siloxene electrodes with the electrolyte ion concentration as follows.

In the normal state,

1 $$\varphi _{(\mathrm {siloxene})} = \varphi ^\circ _{(\mathrm {siloxene})} - \frac{{{{RT}}}}{{{F}}}\ln \frac{X}{{\left( {1 - X} \right)}} - \frac{{{P}}}{{{X}}}$$

When subjected to an applied compressive force,

2 $$\varphi _{(\mathrm {siloxene})} = \varphi ^\circ _{(\mathrm {siloxene})} - \frac{{{{RT}}}}{{{F}}}\ln \frac{{X^\circ }}{{\left( {1 - X^\circ } \right)}} - \frac{{{{P}}^\circ }}{{{{X}}^\circ }}$$

Here, “*φ*_(siloxene)_” represents the actual electrode potential of the two siloxene electrodes in the SCSPC. The first term on the right-hand side, i.e., “*φ*^°^_(siloxene)_”, represents the standard electrode potentials of the two siloxene electrodes used in the SCSPC. The “*R*,” “*T*,” and “*F*” in the second term correspond to the gas constant, temperature, and Faraday constant, respectively. The term “*X*” relates to the activity or occupancy fraction of positive and negatively charged ions at the outer and/or inner surface of the negative and positive electrodes, respectively^42^. The term “*P*” in the third term is related to the intercalation pressure due to the intercalation of ions between the interlayers of siloxene sheets^43^. The terms “*X*^ο”^ and “*P*^ο”^ correspond to the above-mentioned “*X*” and “*P*” terms when subjected to a compressive force. When the siloxene SCSPC is subjected to a compressive force (i.e., under the piezoelectric field), the positive (TEA^+^) and negative (BF_4_^−^) ions move towards the negatrode and positrode in the siloxene SCSPC, which leads to the change in occupancy fraction of ions at the siloxene electrodes (i.e., *X*^ο^ > *X*). This results in the origin of a net potential difference between the two siloxene (energy storage) electrodes in the SCSPC device, which leads to the self-charging process.

**Fig. 8:**
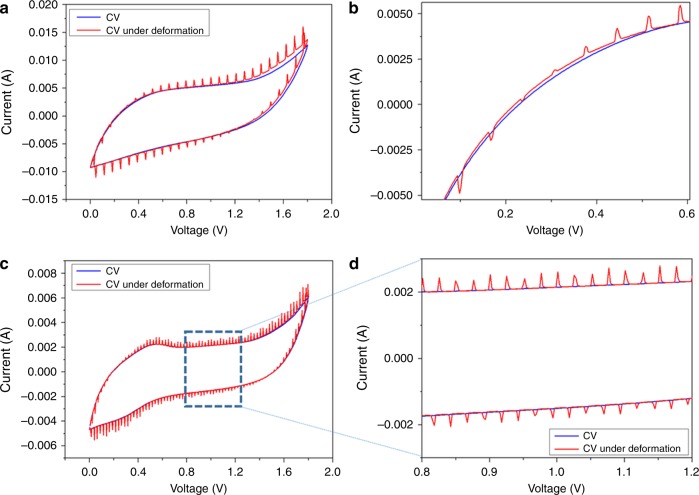
Piezoelectrochemical spectroscopic measurements of siloxene SCSPC. **a** PECS measurement using cyclic voltammetry recorded at a scan rate of 100 mV s^−1^, **b** enlarged portion of Fig. 8a near the PZC region, **c** PECS measurements recorded using a scan rate of 50 mV s^−1^, and **d** represents the enlarged portion of Fig. 8c. The siloxene SCSPC device was subjected to an applied compressive force of 20 N during the PECS measurement.

To understand the piezoelectrochemical process in detail, we used PECS to examine the charge-state dependency of the siloxene SCSPC when subjected to a compressive force^44^. The PECS measurements involve characterizing the cyclic voltammetric profiles (Fig. 8) of the siloxene SCSPC with and without applied compressive forces (mechanical deformation). Figure 8a shows the results of the PECS measurement of the siloxene SCSPC recorded using CV measurements (scan rate of 100 mV s^−1^) over an OVW from 0 to +1.8 V. The comparison of CV profiles with and without mechanical deformation provides significant changes in the magnitude of the current of SCSPC (induced by piezoionic movement) with respect to the applied voltage. In the absence of any compressive force, the CV profile of the siloxene SCSPC shows quasi-rectangular shaped curves, which is similar to the results shown in Fig. 5a. Here, the mechanism of charge storage at the siloxene SCSPC is due to the direct electrochemical process occurring at the electrolyte–electrode interface^23^. The CV profile of the siloxene SCSPC under continuous applied compressive forces shows the emergence of current spikes that are attributed to the additional current generated via the piezoelectrochemical process. When a force acts on the siloxene SCSPC device, the formation of the piezoelectric field at the surface of the separator drifts the electrolyte ions to the siloxene electrode surface. This excessive charge injected from the electrolyte to the electrode surface via the piezoelectrochemical process is observed as current spikes in the CV profile of siloxene SCSPC (subjected to compressive force). The enlarged portion of the PECS results near the potential of zero charge (PZC) region is provided in Fig. 8b, from which it is evident that the current spikes are observed in negative polarity below the PZC region, and the current spikes were observed in positive polarity above the PZC^45^. Figure 8c, d shows the PECS results of the siloxene SCSPC device recorded from CV profiles at a scan rate of 50 mV s^−1^, which shows similar results in comparison with Fig. 8a. The voltage dependency on the magnitude of the piezo-electrochemically generated current can be related to the influence “state of charge (SOC)” of the siloxene SCSPC^46,47^. The increase in the magnitude of the current spikes from the PZC region to high voltage is due to the differential electrolyte ion concentration at various applied voltages^48–51^. This can be directly related to the term “(*P*^ο^/*X*^ο^)” (from Eq. (2)), as explained as follows: The concentration of electrolyte ions near the PZC region is lower compared to that in the increasing applied voltage ranges. When the siloxene SCSPC device is subjected to an applied compressive force, the current spikes are of low magnitude at lower applied potentials due to the low ion concentration at the siloxene electrode surface. At a much higher applied potential, the highly concentrated ions surround the siloxene electrode, which leads to more localized charge injection at their surfaces due to an applied compressive force, thus resulting in the higher magnitude of current spikes. To confirm this, we examined the chronoamperogram of siloxene SCSPC at different applied potentials (0.0, 0.25, 1.5, and 1.8 V) under compressive forces similar to the nanoimpact measurements reported in the literature^52^. The presence of spikes in the negative polarity for applied potential values of 0.0 and 0.25 V was observed from Supplementary Fig. 11a, b. When the applied potential values are increased to 1.5 and 1.8 V, the polarity of the piezo-induced generated current changed, as evident from Supplementary Fig. 11c, d. Furthermore, the effect of applied compressive forces is presented in Supplementary Fig. 12, which indicates the significant role of the piezoelectrochemical process on the generated current through excessive charge injection from the electrolyte to the siloxene electrodes. Altogether, the PECS measurements directly confirm the “piezoelectrochemical effect” involved in the self-charging process of the siloxene SCSPC, which is of great benefit to understand the mechanism of energy conversion and storage in SCSPC.

## Discussion

The significant findings of this study realized the use of PECS measurements to directly probe the “piezoelectrochemical effect” involved in the mechanism of energy conversion and storage in the siloxene SCSPC. This approach is very important for understanding the mechanism of the piezoelectrically driven electrochemical process involved in SCSPC. The siloxene SCSPC can directly convert mechanical energy into electrical energy and store it in a single integrated device with better self-charging properties (superior to many of the reported SCSPC) and good electromechanical stability. Considering the achieved self-charging performance and demonstrated real-time application of the siloxene SCSPC with direct evidence of the piezoelectrochemical effect (using PECS analysis), we ensure that this work will provide a road map towards the development of next-generation piezoelectrically driven SCSPC.

## Methods

### Materials

Calcium silicide (CaSi_2_) powders were obtained from Kojundo Chemicals Laboratory Co., Ltd, Japan. The polyvinylidene fluoride (PVDF) powders were purchased from Sigma Aldrich Ltd, South Korea. Hydrochloric acid (HCl), acetone and dimethylacetamide, and *N*-methyl-2-pyrrolidone (NMP), were purchased from Dae-Jung Chemicals Ltd, South Korea. The tetraethylammonium tetrafluoroborate (TEABF_4_) electrolyte was purchased from Alfa Aesar Chemicals, South Korea.

### Preparation of siloxene sheets

The siloxene sheets was prepared via topochemical route, which involves the deintercalation of calcium from CaSi_2_ in the presence of ice-cold HCl^22,23^. Briefly, CaSi_2_ powders (1 g) were slowly added to the solution containing conc. HCl (100 mL) at 0 °C and allowed to vigorously stir via a magnetic stirrer for 4 days. A transformation of color from grayish black (CaSi_2_) to green (siloxene sheets) indicated the dissolution of calcium in the HCl solution, which led to the formation of siloxene sheets. After the termination of the reaction, the green-colored siloxene powders were washed with water and acetone. Furthermore, the prepared siloxene powder was redispersed in water (100 mL) and allowed to ultrasound irradiation for 1 h, followed by a similar washing process. Finally, the prepared siloxene sheets were dried at 80 °C for 12 h.

### Electrospinning of the siloxene–PVDF piezofiber separator

The siloxene-incorporated PVDF piezofibers were fabricated by the electrospinning process^17^. Here, the PVDF solution was obtained dissolving PVDF powders (0 wt%) in a 30:70 (v/v) ratio of acetone and dimethylacetamide using stirring process until a homogeneous viscous solution was formed. After that, the siloxene sheet (10 wt%) was added into the PVDF solution in the presence of ultrasound irradiation process for 2 h that resulted in the formation of a homogeneous dispersion of siloxene sheets in PVDF solution. The obtained viscous solution containing siloxene and PVDF was loaded into a syringe (15 mL) with a stainless-steel nozzle (21G). Then, the electrospinning process was carried out at a constant flow rate of 500 µL h^−1^ under a constant DC voltage of 15 kV. Here, the distance between the tip to the collector is kept at 10 cm to obtain a siloxene/PVDF fibrous mat. Finally, the obtained siloxene/PVDF piezofiber separator was dried at 60 °C for 12 h.

### Instrumentation

The ultrasound irradiation process was carried out using an ultrasonic processor (Model No: VCX 750, Sonics and Materials, Inc., USA (750 W, 20 kHz)) with a titanium horn. The electrospinning process for the preparation of siloxene/PVDF piezofibers was carried out on NanoNC electrospinning instrument (Model: ESR200R2, South Korea). The X-ray diffractogram of siloxene sheets was recorded using an Empyrean X-ray diffractometer (Malvern Panalytical, UK) with Cu-Kα radiation (*λ* = 1.54184 Å). The Fourier transform infrared spectrum (FT-IR) was measured using a Thermo Scientific Nicolet-6700 FT-IR spectrometer. The laser Raman spectra were obtained using a Lab Ram HR Evolution Raman spectrometer (Horiba Jobin-Yvon, France, at a laser excitation source of wavelength 514 nm). The chemical state of elements present in the siloxene sheets was analyzed by an X-ray photoelectron spectrometer (ESCA-2000, VG Microtech Ltd). The surface morphology of the siloxene powders and electrospun fibers was examined using field emission scanning electron microscopy (TESCAN, MIRA3) under different magnifications with energy dispersive X-ray spectroscopy (EDS) and HR-TEM (JEM-2011, JEOL) with a CCD 4k × 4k camera (Ultra Scan 400SP, Gatan).

### Fabrication of the siloxene-based SCSPC device

The energy storage electrodes in this work were fabricated by dispersing appropriate siloxene and PVDF powders in a weight ratio of 95:5 in NMP solvent and allowed to sonicate for 30 min. After this, the viscous ink (containing siloxene:PVDF in NMP) was coated onto the surface of carbon fabric via drop-casting and dried at 80 °C for 12 h. The preparation of the TEABF_4_ ionogel was reported in our earlier work^17^. The siloxene SCSPC device was fabricated in the form of SSCs by sandwiching the siloxene–PVDF piezofiber separator (gelled with TEABF_4_ ionogel) between two siloxene-coated carbon fabrics. Finally, the fabricated SCSPC device is sealed with a moisture-resistant pouch using a GBC Fusion 3000L Laminator. The preparation of ionogel electrolyte and fabrication of siloxene SCSPC device were performed in a glove box filled with argon.

### Electrical and electrochemical analysis

The energy-harvesting properties of the siloxene/PVDF piezofiber mat with metallic aluminum foil as the top and bottom electrodes (sealed using a moisture-resistant pouch) were measured under various levels of applied compressive forces with the use of LinMot E1100 linear motor. The output voltage was recorded by a Keithley Electrometer (Model no: 6514). The energy storage and self-charging properties of the siloxene SCSPC were measured on an Autolab PGSTAT302N electrochemical workstation. The electrochemical analysis such as CV recorded using different levels of applied scan rates, galvanostatic CD recorded using various applied current ranges, EIS, and long-term cyclic stability tests were used to understand the charge-storage performance of the siloxene SCSPC. The mechanical flexibility of the siloxene SCSPC was studied by measuring its CV profiles in the bent state using a bending tester machine (JUNIL-JIBT-200). The self-charging properties of the siloxene SCSPC were determined by measuring the voltage (using an electrochemical workstation) while subjecting the device to continuous applied compressive force. A constant discharge current is applied to the siloxene SCSPC to discharge after the self-charging process. The specific device capacitance, energy (*E*) and power (*P*) density of the siloxene SCSPC devices were determined from the CD profiles using the following relations^26,53^:

3 $${{C}}_{\mathrm{A}} = ({{I}} \times {\mathrm{T}}_{\mathrm{d}})/\left( {{{A}} \times \Delta {{V}}} \right)$$

4 $${{E}} = \left[ {{{C}}_{\mathrm{A}} \times \Delta {{V}}^2} \right]/2$$

5 $${{P}} = {{E}}/{{T}}_{\mathrm{d}}$$

Here, *C*_A_ represents the specific areal capacitance (F cm^−2^), *I* is the discharge current, *T*_d_ is the time required for discharge, *A* is the electroactive area of the device, and Δ*V* is the OVW.

## Supplementary information

Supplementary Information

## Data Availability

The authors declare that the data supporting the findings of this study are available within the paper [and its supplementary information files]. The data that support the findings of this study are available from the corresponding author upon reasonable request.
