# Supplementary material for: Probing the energy conversion process in piezoelectric-driven electrochemical self-charging supercapacitor power cell using piezoelectrochemical spectroscopy
Source: Nat Commun. 2020 May 11;11:2351. doi: 10.1038/s41467-020-15808-6 (PMC7214414; doi:10.1038/s41467-020-15808-6)
Supplement: Supplementary file 1 — Supplementary Information [file 41467_2020_15808_MOESM1_ESM.pdf]

Supplementary information

Probing the energy conversion process in piezoelectric-driven electrochemical self-charging supercapacitor power cell using piezoelectrochemical spectroscopy

Karthikeyan Krishnamoorthy[1#], Parthiban Pazhamalai[1#], Vimal Kumar Mariappan[1], Swapnil Shital Nardekar[1], Surjit Sahoo[1], Sang-Jae Kim[1,2*]

[1]Nanomaterials and System Laboratory, Major of Mechatronics Engineering, Faculty of Applied Energy system, Jeju National University, Jeju 63243, South Korea.

[2]Department of Advanced Convergence Science & Technology, Jeju National University, Jeju 63243, South Korea.

Corresponding author Email: kimsangj@jejunu.ac.kr

[#] These authors contributed equally (Karthikeyan Krishnamoorthy and Parthiban Pazhamalai).

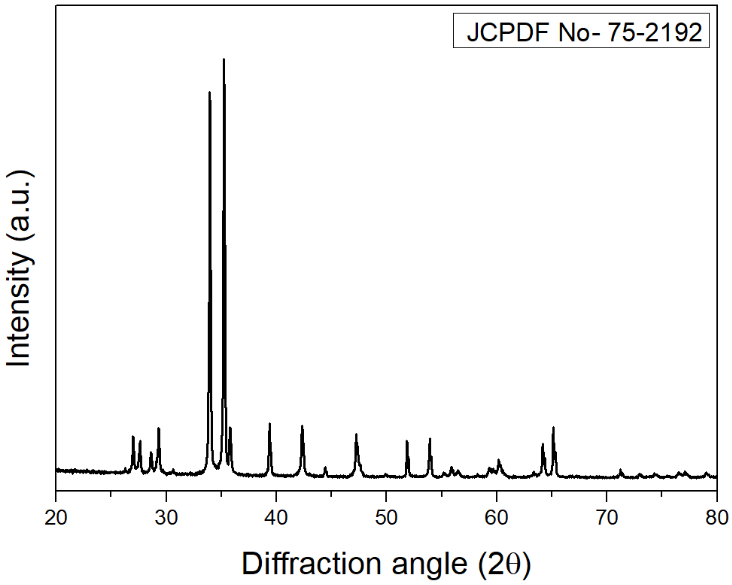

Supplementary Figure 1. X-ray diffraction pattern of $CaSi_2$.

Supplementary Figure 1 represents the X-ray diffraction pattern of calcium silicide powders (precursor used for the preparation of siloxene sheets). The diffraction pattern of the $CaSi_2$ powders closely matched with the JCPDF file no-75-2192.

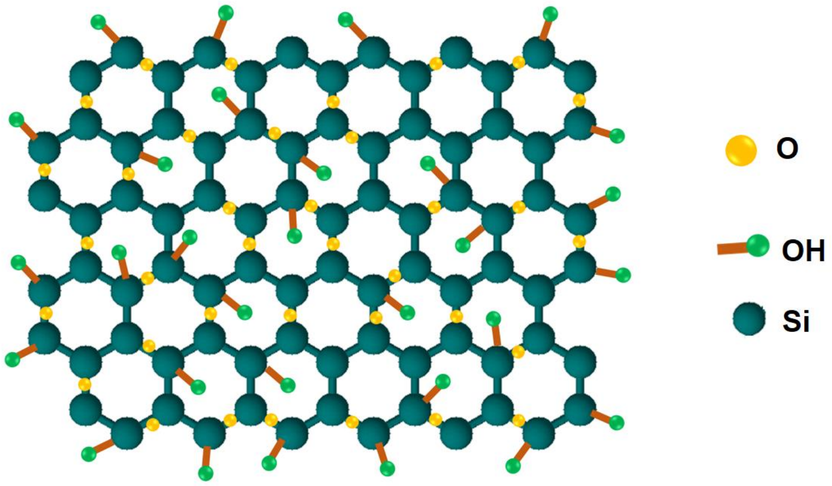

Supplementary Figure 2. Structure of siloxene sheets prepared via topochemical de-intercalation of calcium from $CaSi_2$.

Supplementary Figure 2 shows the molecular structure of siloxene sheets, which is of Kautsky-type based on the results of FT-IR spectra (given in Figure 2(C) of the main manuscript). It represents the presence of hexagonal silicon rings interconnected via Si–O–Si bridges in the silicon planes with some of the oxygenated functional groups bonded at the edges and basal planes of the silicon sheets [1–3].

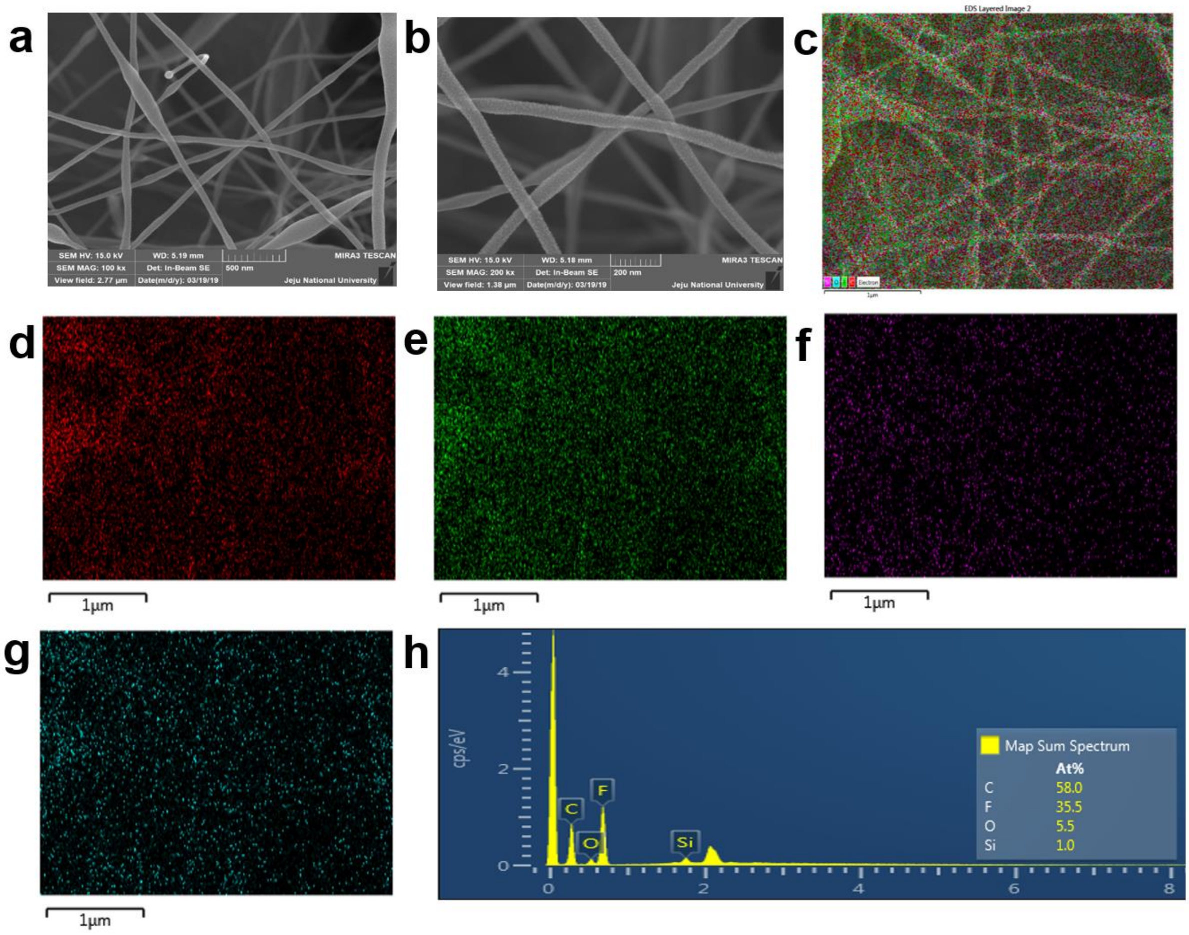

Supplementary Figure 3. **a-b** Field-emission scanning electron micrograph of siloxene-PVDF piezofiber at low **a** and high **b** magnification, **c** Overlay field emission micrograph of siloxene-PVDF piezofiber; **d-g** EDX mapping of **d** carbon, **e** fluorine, **f** silicon and **g** oxygen atoms present in siloxene-PVDF piezofibers, and **h** EDX mapping of siloxene-PVDF piezofiber.

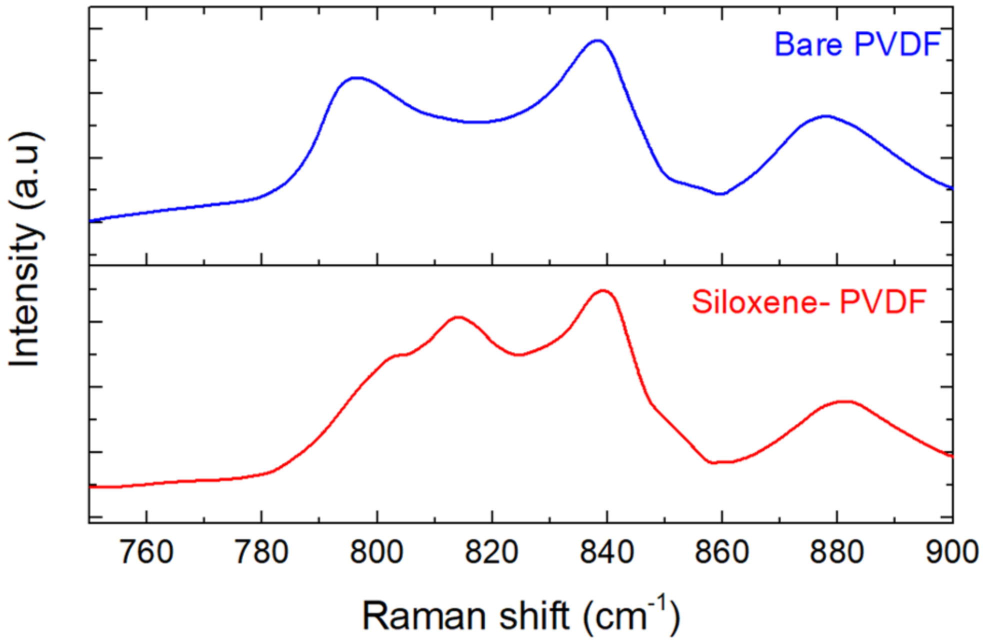

Supplementary Figure 4. Laser Raman spectra of bare PVDF and siloxene-PVDF electrospun piezofiber.

Supplementary Figure 4 shows the laser Raman spectra of bare electrospun PVDF and siloxene-PVDF piezofibers over the region 780 to 900 cm^{-1}. The Raman spectrum of bare electrospun PVDF shows three major bands at 796, and 838 cm^{-1} which corresponds to the *α, and β* phase of PVDF, respectively[4,5]. The band observed at 878 cm^{-1} arised due to the combination of vibrations from CF_2 groups and carbon-carbon skeleton of the PVDF[6]. The Raman spectrum of siloxene-PVDF fiber shows the presence of vibrational bands at 802, 814, 839, and 879 cm^{-1}, respectively. The presence of the band at 802 cm^{-1} is due to the *α* phase of PVDF, and the observed small shift in the band position is due to the interaction between siloxene sheets and PVDF. The band observed at 814 cm^{-1} in the electrospun siloxene-PVDF might be originated either from the Si-O-Si present in the siloxene sheets or *γ* phase of PVDF [3,7,8]. Further, the characteristic bands of PVDF, such as the *β* phase and vibrations of CF_2 and carbon-carbon chains, were preserved in the spectrum of siloxene-PVDF fiber.

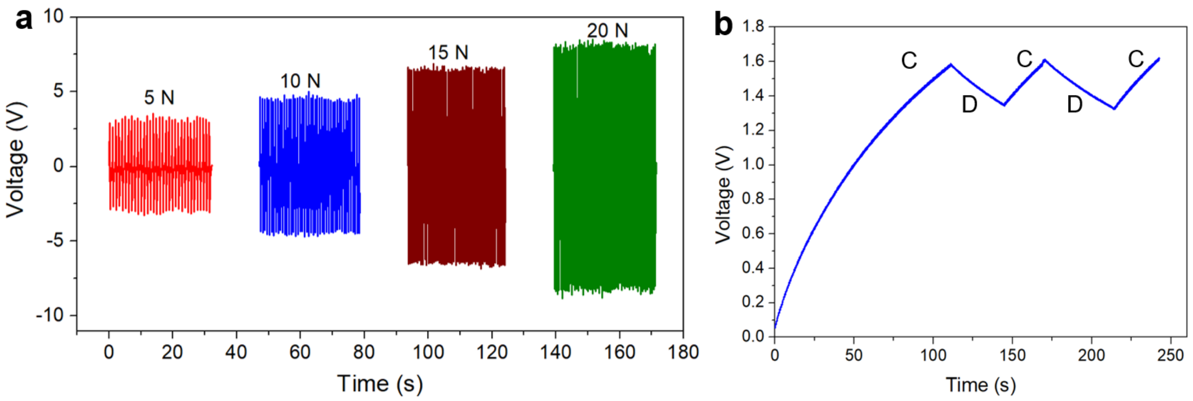

Supplementary Figure 5 **a** Mechanical energy harvesting properties of siloxene-PVDF piezofiber obtained using various levels of compressive forces, and **b** Charging of commercial capacitor (0.22 μF) using electrospun siloxene-PVDF piezofiber.

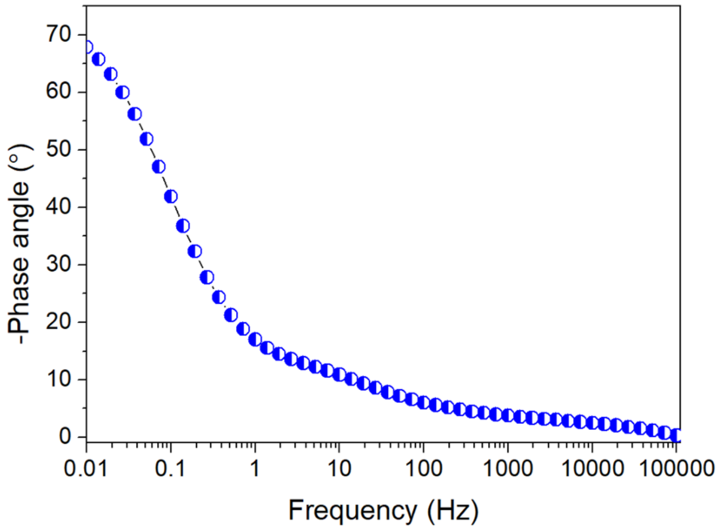

Supplementary Figure 6. Bode phase angle plot of the siloxene SCSPC device.

Supplementary Figure 6 shows the Bode phase angle plot of siloxene SCSPC, which shows that the phase angle at the low-frequency region is about -69°, thus indicating the pseudocapacitive nature of the siloxene electrode [2,9].

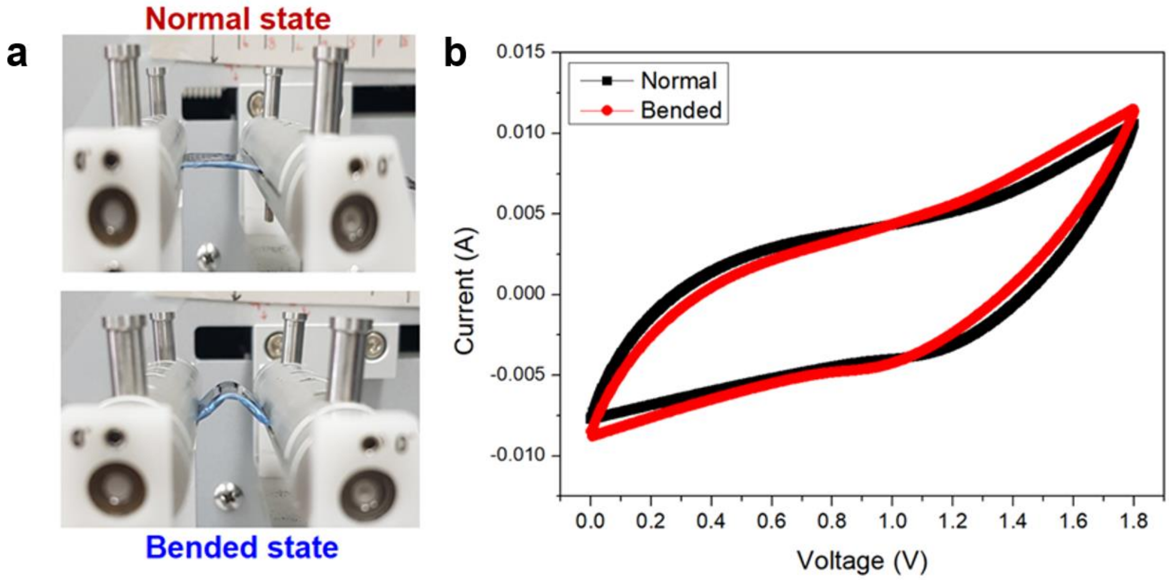

Supplementary Figure 7. Mechanical flexibility studies of siloxene SCSPC tested via bending the device. **a** Represents the digital photographs of siloxene SCSPC at normal and bended states, respectively, and **b** represents the CV profiles of siloxene SCSPC obtained at normal and bended conditions recorded using a scan rate of 100 mV s^{-1}.

The mechanical flexibility of the siloxene SCSPC was studied by measuring their CV profiles at normal and bended states (as shown in Supplementary Figure **7a**). The shape of the CV profiles in Supplementary Figure **7b** is almost similar for both the normal and bended states, which indicated the better mechanical flexibility of the SCSPC. The retention of specific device capacitance (C/C_o) obtained at bended condition (C) to that of normal condition (C_o) is found to be 1.07, which highlights the mechanical stability of the siloxene SCSPC. The observed small difference in the capacitance values might be due to the changes in the electrochemically active area[10] and strain-induced piezo-ionic/mechano-ionic properties of the ionogel[11,12] used in the siloxene SCSPC. Overall, these studies indicated that the siloxene SCSPC possesses excellent mechanical flexibility.

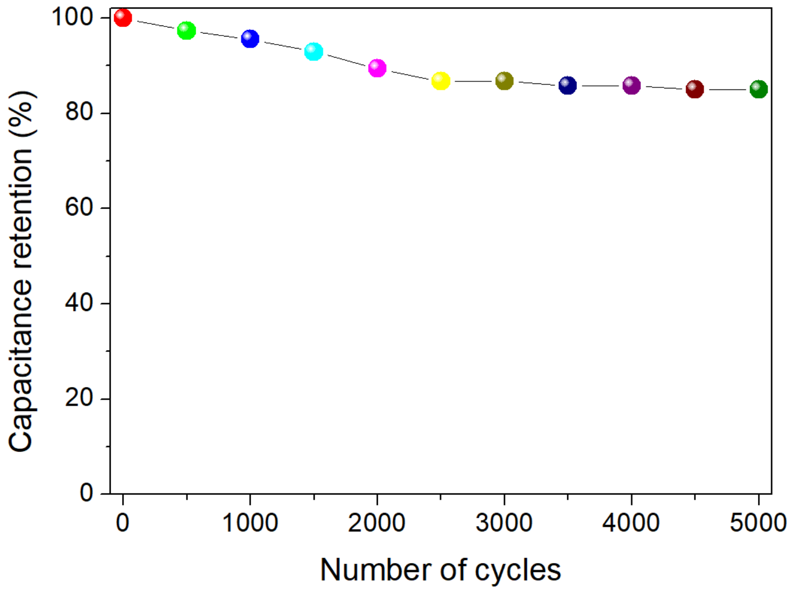

Supplementary Figure 8. Long-term cyclic stability of SCSPC device over 5000 consecutive cycles of charge-discharge using an applied current of 10 mA.

Supplementary Figure 8 shows the long term cyclic stability of the siloxene SCSPC over 5000 continuous CD measurements (using an applied current of 10 mA), which showed better capacitance retention of 85 %, highlighting the superior electrochemical stability of the siloxene SCSPC[13]. The capacitance retention values are comparable and/or higher compared to the state of art of supercapacitors such as rGO SSC (93% after 1500 cycles)[14], RGOCMK-5 composite SSC (90% after 2000 cycles)[15], and RGO-RuO_2 SSC (70% after 2000 cycles)[16].

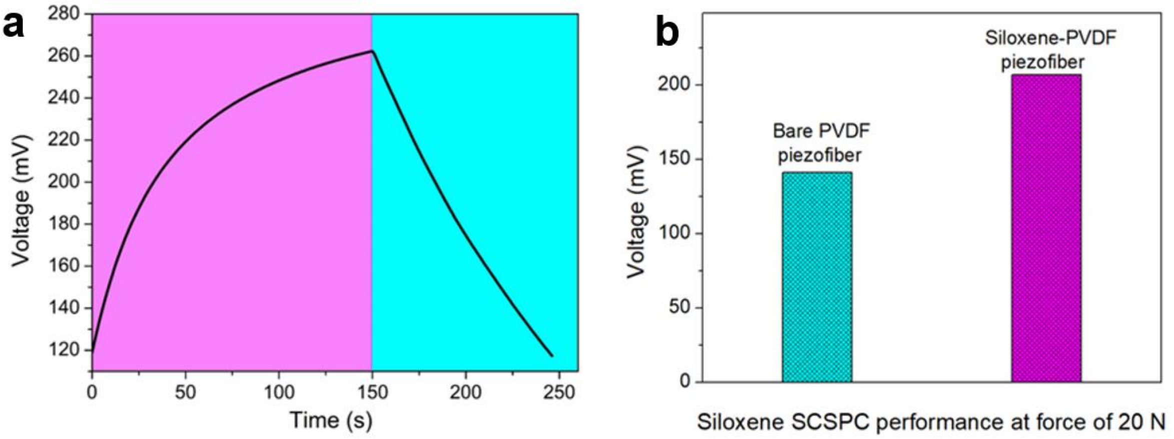

Supplementary Figure 9. **a** Self-charging and discharge properties of siloxene SCSPC fabricated using bare electrospun PVDF fibers subjected to a compressive force of 20 N, and **b** Comparative self-charging performance of siloxene SCSPC fabricated using bare PVDF and siloxene-PVDF piezofibers.

Supplementary Figure **9a** presents the self-charging properties of the siloxene SCSPC fabricated using bare electrospun PVDF piezofibers. When this device was subjected to an applied compressive force of 20 N, the device was able to self-charge upto 141 mV (from 118 mV into 259 mV) followed by discharging using a constant current of 10 μA, respectively. At the same conditions, the siloxene SCSPC device fabricated using siloxene-PVDF piezofibers was able to charge upto 207 mV (See Figure **6c**) in the main article and Supplementary Figure **9b**). This study indicated the better self-charging performance of the siloxene SCSPC fabricated using siloxene-PVDF piezofibers over the bare PVDF piezofibers. This is due to the higher mechanical to electrical transduction efficiency of the siloxene-PVDF piezofibers that leads to high piezopotential than the bare PVDF piezofibers, as seen in Figure **4a** in the main article. The obtained results are in good agreement with the previous studies on SCPCs devices using modified PVDF separators demonstrated in the literature[17,18].

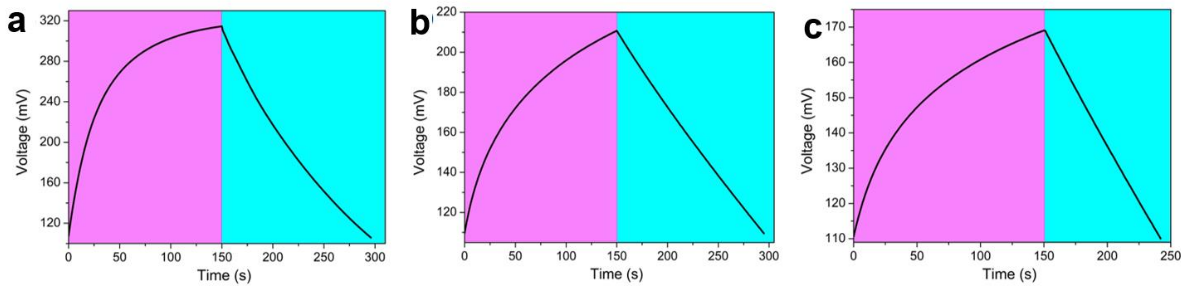

Supplementary Figure 10. Self-charging and discharge properties of siloxene SCSPC under different frequencies [**a** 2 Hz, **b** 1 Hz, **c** 0.5 Hz] at a force of 20 N, respectively. The discharge current is 20, 10, and 10 μA for SCSPC subjected to 2, 1, and 0.5 Hz, respectively.

Supplementary Figure 10 shows the effect of the applied frequency of the compressive force (20 N) on the self-charging performances of siloxene SCSPC. When the frequencies were 2, 1, and 0.5 Hz under an applied compressive force of 20 N, the self-charging performances of the siloxene SCSPC was found to be 207, 102, and 59 mV, respectively. A higher frequency results in an increased self-charging voltage of the siloxene SCSPC that is due to a higher input power of the siloxene-PVDF piezofiber at a higher frequency. This finding is in close agreement with the previous works on the integrated SCPCs.[17–19]

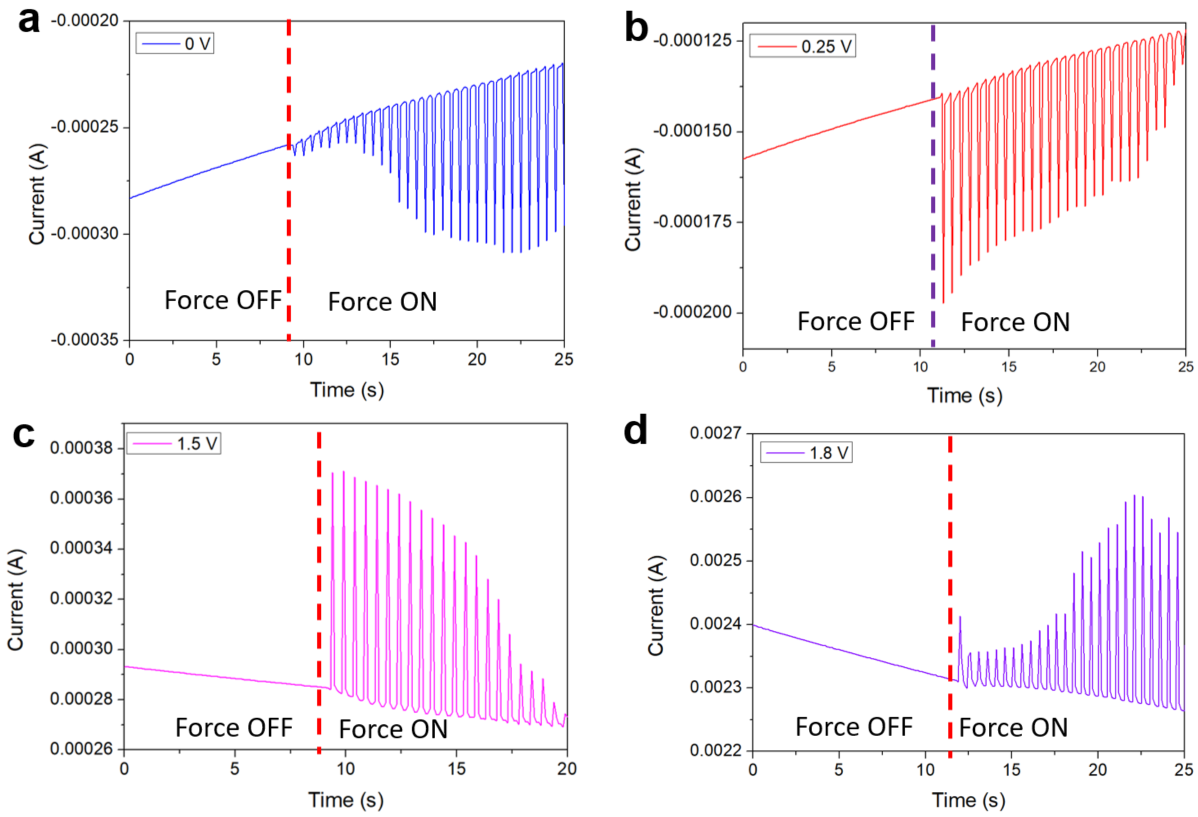

Supplementary Figure 11. Piezoelectrochemical spectroscopy of siloxene SCSPC recorded using chronoamperometry with different applied potentials such as **a** 0.0 V, **b** 0.25 V, **c** 1.5 V and **d** 1.8 V, respectively.

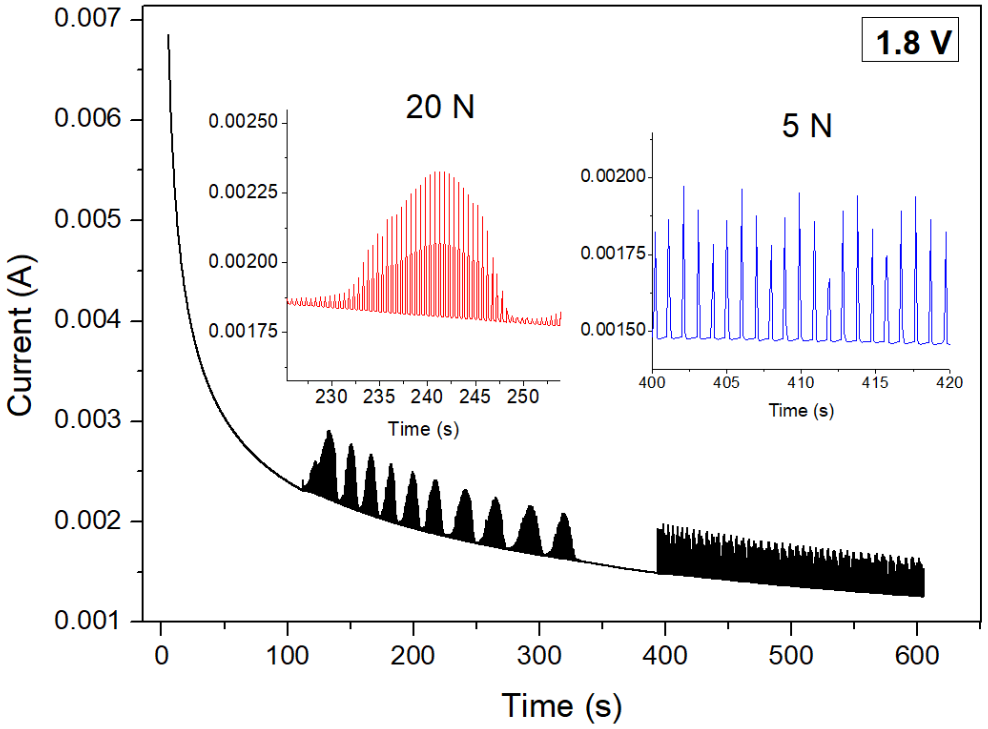

Supplementary Figure 12. Piezoelectrochemical spectroscopy of siloxene SCSPC recorded using chronoamperometry with an applied potential of 1.8 V. A compressive force of 20 and 5 N were provided to the siloxene SCSPC device from 100 to 340 seconds, and 380 to 600 seconds.

Supplementary Table 1: Performance metrics of siloxene SCSPC with reported silicon based SSCs.

S. No	Electrode material	Electrolyte	Specific capacitance	Energy density	Power density	Ref
1.	Silicon Nanowire	$PYR_{13}TFSI$	30 μF/cm^2	0.19 mJ / cm^2	1 to 2 mW/cm^2	20
2.	Silicon Nanotrees	EMI-TFSI	3.8 mF/cm^2	2.8 mJ/cm^2	2 mW/cm^2	21
3.	Diamond coated Si NWs	PMPyrrTFSI	105 μF/cm^2	84 μJ/cm^2	0.94 mW/cm^2	22
4.	Silicon Nanotrees	EMI-TFSI	58.3 μF/cm^2	0.262 mJ/cm^2	–	23
5.	Porous Silicon	$PEO-EMIBF_4$	3.5 mF/cm^2	0.17 μWh/cm^2	22 μW/cm^2	24
6.	Oxide coated Si	EMI-TFSI	31 μF/cm^2	0.212 mJ/cm^2	0.472 mW/cm^2	25
7.	PEDOT coated Silicon nanowires	$PYR_{13}TFSI$	8-9 mF/cm^2	9 mJ/cm^2	0.8 mW/cm^2	26
8.	Graphene coated Silicon Nanowires	EMIM][N(Tf)2	0.24 mF/cm^2	–	–	27
9.	$MoSe_2$ SCSPC	$TEABF_4$ Ionogel	18.93 mF/cm^2	37.9 mJ/cm^2	0.268 mW/cm^2	5
10.	Siloxene SCSPC	$TEABF_4$ Ionogel	28.98 mF/cm^2	46.97 mJ/cm^2	1.12 mW/cm^2	This work

Note: 1. (–) indicates that the values are not provided in the corresponding references.

Supplementary References

1. Fu, R. *et al.* Two-dimensional silicon suboxides nanostructures with Si nanodomains confined in amorphous SiO 2 derived from siloxene as high performance anode for Li-ion batteries. *Nano Energy* **39**, 546–553 (2017).
2. Krishnamoorthy, K., Pazhamalai, P. & Kim, S. J. Two-dimensional siloxene nanosheets: Novel high-performance supercapacitor electrode materials. *Energy Environ. Sci.* **11**, 1595–1602 (2018).
3. Pazhamalai, P., Krishnamoorthy, K., Sahoo, S., Mariappan, V. K. & Kim, S. J. Understanding the Thermal Treatment Effect of Two-Dimensional Siloxene Sheets and the Origin of Superior Electrochemical Energy Storage Performances. *ACS Appl. Mater. Interfaces* **11**, 624–633 (2019).
4. Xu, F. *et al.* Facile preparation of highly oriented poly(vinylidene fluoride) uniform films and their ferro- and piezoelectric properties. *RSC Adv.* **7**, 17038–17043 (2017).
5. Pazhamalai, P. *et al.* A High Efficacy Self-Charging MoSe2 Solid-State Supercapacitor Using Electrospun Nanofibrous Piezoelectric Separator with Ionogel Electrolyte. *Adv. Mater. Interfaces* **5**, 1800055 (2018).
6. Hilczer, B. *et al.* Properties of PVDF-MCM41 Nanocomposites Studied by Dielectric, Raman and NMR Spectroscopy. *Ferroelectrics* **472**, 64–76 (2014).
7. Singh, P., Borkar, H., Singh, B. P., Singh, V. N. & Kumar, A. Ferroelectric polymer-ceramic composite thick films for energy storage applications. *AIP Adv.* **4**, 087117 (2014).
8. Yamanaka, S., Matsu-ura, H. & Ishikawa, M. New deintercalation reaction of calcium from calcium disilicide. Synthesis of layered polysilane. *Mater. Res. Bull.* **31**, 307–316 (1996).
9. Ren, G., Li, S., Fan, Z.-X., Hoque, M. N. F. & Fan, Z. Ultrahigh-rate supercapacitors

with large capacitance based on edge oriented graphene coated carbonized cellulous paper as flexible freestanding electrodes. *J. Power Sources* **325**, 152–160 (2016).

10. Li, W. *et al.* Mechanically robust 3D hierarchical electrode via one-step electro-codeposition towards molecular coupling for high-performance flexible supercapacitors. *Nano Energy* 104275 (2019). doi:10.1016/j.nanoen.2019.104275

11. Dobashi, Y., Allegretto, G., Sarwar, M. S., Cretu, E. & Madden, J. D. W. Mechanoionic Transduction of Solid Polymer Electrolytes and Potential Applications. *MRS Adv.* **1**, 63–68 (2016).

12. Villa, S. M. *et al.* Soft Piezoionic/Piezoelectric Nanocomposites Based on Ionogel/BaTiO 3 Nanoparticles for Low Frequency and Directional Discriminative Pressure Sensing. *ACS Macro Lett.* **8**, 414–420 (2019).

13. Hu, X., Zhang, W., Liu, X., Mei, Y. & Huang, Y. Nanostructured Mo-based electrode materials for electrochemical energy storage. *Chem. Soc. Rev.* **44**, 2376–2404 (2015).

14. Liu, W., Yan, X., Lang, J., Peng, C. & Xue, Q. Flexible and conductive nanocomposite electrode based on graphene sheets and cotton cloth for supercapacitor. *J. Mater. Chem.* **22**, 17245 (2012).

15. Lei, Z. *et al.* A high-energy-density supercapacitor with graphene–CMK-5 as the electrode and ionic liquid as the electrolyte. *J. Mater. Chem. A* **1**, 2313 (2013).

16. Zhang, J., Jiang, J., Li, H. & Zhao, X. S. A high-performance asymmetric supercapacitor fabricated with graphene-based electrodes. *Energy Environ. Sci.* **4**, 4009 (2011).

17. Xue, X. *et al.* CuO/PVDF nanocomposite anode for a piezo-driven self-charging lithium battery. *Energy Environ. Sci.* **6**, 2615 (2013).

18. Xing, L., Nie, Y., Xue, X. & Zhang, Y. PVDF mesoporous nanostructures as the piezo-separator for a self-charging power cell. *Nano Energy* **10**, 44–52 (2014).

19. Xue, X., Wang, S., Guo, W., Zhang, Y. & Wang, Z. L. Hybridizing Energy Conversion

and Storage in a Mechanical-to-Electrochemical Process for Self-Charging Power Cell. *Nano Lett.* **12**, 5048–5054 (2012).

20. Aradilla, D. *et al.* High performance of symmetric micro-supercapacitors based on silicon nanowires using N-methyl-N-propylpyrrolidinium bis (trifluoromethylsulfonyl) imide as electrolyte. *Nano Energy* **9**, 273–281 (2014).
21. Eftekhari, A. Supercapacitors utilising ionic liquids. *Energy Storage Mater.* **9**, 47–69 (2017).
22. Gao, F. *et al.* Diamond-coated silicon wires for supercapacitor applications in ionic liquids. *Diam. Relat. Mater.* **51**, 1–6 (2015).
23. Thissandier, F., Gentile, P., Brousse, T., Bidan, G. & Sadki, S. Are tomorrow's micro-supercapacitors hidden in a forest of silicon nanotrees? *J. Power Sources* **269**, 740–746 (2014).
24. Cohn, A. P. *et al.* All Silicon Electrode Photocapacitor for Integrated Energy Storage and Conversion. *Nano Lett.* **15**, 2727–2731 (2015).
25. Berton, N. *et al.* Wide-voltage-window silicon nanowire electrodes for micro-supercapacitors via electrochemical surface oxidation in ionic liquid electrolyte. *Electrochem. commun.* **41**, 31–34 (2014).
26. Aradilla, D. *et al.* Novel hybrid micro-supercapacitor based on conducting polymer coated silicon nanowires for electrochemical energy storage. *RSC Adv.* **4**, 26462 (2014).
27. Soam, A., Kavle, P., Kumbhar, A. & Dusane, R. O. Performance enhancement of micro-supercapacitor by coating of graphene on silicon nanowires at room temperature. *Curr. Appl. Phys.* **17**, 314–320 (2017).
